# A bibliometric analysis using a newly developed model and a customizable research tool: A case study of researcher mobility in Sweden

**DOI:** 10.1371/journal.pone.0308147

**Published:** 2024-12-02

**Authors:** Silvia Dobre, Rachel Herbert, Alvin Shijie Ding, Hans Pohl

**Affiliations:** 1 International Center for the Study of Research, Elsevier, Oxford, United Kingdom; 2 International Center for the Study of Research, Elsevier, Beijing, China; 3 STINT, the Swedish Foundation for the Internationalisation of Research and Higher Education, Stockholm, Sweden; 4 Chalmers University of Technology, Gothenburg, Sweden; Lund University, SWEDEN

## Abstract

Researcher mobility is an integral part of the way research is conducted and of a researcher’s career. Its effects on collaboration networks, research impact and knowledge flows drive countries and institutions to quantify and understand this activity. The purpose of this study is to test a new researcher mobility model which was developed and prototyped as a customisable research tool to provide a unified perspective on mobility at macro (national), meso (institutional) and micro (individual) levels. The approach includes multidimensional perspectives, including temporal, geographical, sectoral, directional mobility, that could be used for benchmarking and trend analyses. The model quantifies research mobility volumes and qualifies the mobility flow additional researcher characteristics and productivity indicators. We tested the tool among Sweden’s higher education sector, observing researcher mobility patterns between 1992–2021. Results show a high degree of variability in researcher mobility patterns across institutions, especially when considered by career age. Larger higher education institutions in Sweden tend to see a high level of inter-university mobility: most of the Outflow researchers have international mobility and were affiliated with organisations from diverse sectors. Smaller universities are more adapted to attract early- and retain late-career researchers. One university was identified as an incubator for early-career researchers that go on to high levels of mobility. Another university achieved higher mobility rates by facilitating short-term mobility abroad. The study highlighted a shift in the countries of destination for the Inflow early-career researchers: fewer were affiliated with USA, UK or Japan, while other countries became more prominent (China, Germany, Netherlands, Spain) and new destinations emerged (Brazil, India, Iran). The study emphasized that visiting researchers are consistently more productive, and their research impact is generally higher. With the help of our advanced model, we present a detailed picture of mobility in Sweden and demonstrate the power of this customisable tool.

## Introduction

Mobility is an integral part of the way research is conducted. In many cases, mobility is essential for the researcher, though the reasons can vary: a change of institution could allow field studies to be carried out, enable access to unique infrastructure or research funding, or be in response to a job opportunity, either in the capacity of researcher or lecturer.

Mobility is a useful approach to extend a researcher’s network and create or extend ‘chain-like’ collaborations [1]. This can have powerful effects, with mobile and collaborative researchers tending to have higher rates of research impact [1–3]–although this has been found to differ by gender [3]–and better access to research funding [4] than others.

Mobility can also be considered at a global level and as a mechanism which allows for the sharing and distribution of knowledge between countries, organizations and people. The international nature of research and the global nature of grand challenges drive the international circulation of people and knowledge. Even so, individual countries maintain a focus on the flows of researchers in, out, and within. Indeed, several countries have employed policies to encourage researchers who were domestically trained to return to the country following stints of international mobility [5, 6]. Additionally, there is a desire to understand domestic or national mobility with some countries introducing policies to encourage inter-university movement of researchers [7, 8]. Mobility between academic and corporate actors is also promoted with the intention to foster innovation.

For all these reasons, mobility is promoted at country level [9] and in many cases is required as a part of a researcher’s career. Against this background, it is important to understand if and how the mobility of researchers contributes to the societal value of investments in research. Some studies have been made of the impact of mobility on research which is a part of the overall picture. This study will focus on the relationship between mobility and various indicators based on scientific publications, all the while considering the varied types of mobility (national to international, and sectoral) and distinct levels of aggregation, from an individual institution to a country.

### Background

Prior work has established that a bibliometric approach to tracking researcher mobility is broadly dependable, providing care is taken around the interpretation of the findings and with a recommendation to consider supplementary data [10]. Indeed, Scopus, which is among the largest curated abstract and citation databases [11] is used to track the mobility of individual researchers, which means that data for several decades are available for study.

In part because of this, there have been many studies on the topic of researcher mobility. Among the studies published, many focus on international mobility [12] at global, regional or country level and use, for example, Scopus or Web of Science data often refined with disambiguation algorithms to make the affiliations more reliable [1, 13–16]. Despite this focus, the studies tend to differentiate between researchers demonstrating international or national mobility, and those who are not mobile, though the terminology varies to a degree depending on the perspective [17–19].

The studies centre on varied patterns and implications of mobility, for example the intersection between mobility and collaboration [20]. The international mobility was studied [2] by examining articles published in the American Physical Society journals to identify the profiles of prominent researchers. A similar dataset was used to study mobility between institutions [21], mainly in the United States. In another study [22] ORCID and Carnegie Classification data were used to study the mobility of professors between institutions in the United States. Other approaches to study mobility include the use of CV data [4] and the use of an Italian database comprising personnel at Italian universities [23].

Beyond the traditional views of mobility between research organizations, studies on the mobility of researchers between sectors–particularly industry and academia–often have a goal to encourage such movement and increase the understanding and innovation that might come of the connections [24, 25].

Despite the wealth of literature available on the topic of mobility, the authors believe there is space yet for models or tools to encompass the different facets of mobility. In comparison to previous studies, this study outlines and tests methods to analyse researcher mobility between academic institutions as well as non-academic ones. This with both national and international and cross-sectoral perspectives.

To test the model, Sweden is used as case study: a medium-sized country in terms of research, which has extensive international collaboration [26] and a high proportion of publications co-authored between academic and corporate actors [27]. Yet, there are very few studies on the researcher mobility patterns in Sweden in the academic literature, and most consider specific aspects of mobility such as student mobility [28] or mobility among history researchers [29]. Several studies have looked at sectoral mobility in connection with innovation: however, one study [30] found no instances of staff mobility for the duration of the government funded projects while another [31] reported on the challenges around the movement between the sectors, especially in the case of small and medium size enterprises. However, organizations such as the Swedish Foundation for International Cooperation in Research and Higher Education (STINT) make available country-level reports on mobility, including Sweden [32] using data from Elsevier, but included only mobility in an academic setting [33].

Thus, the academic literature is missing a holistic understanding of Sweden’s mobility patterns, and more broadly, researcher mobility research could be enhanced by incorporating the many different types of mobility from one consistent data source as an approach to trend analysis and benchmarking. Given the many and different perspectives which can be studied using researcher mobility data, the broader and ambitious long-term goal of this work is to develop a tool available for everyone who is interested in the study of researcher mobility.

This study sets out to explore in detail the questions explicitly from those in universities who are connected to and interested in research mobility. Then the project intends to develop an innovative approach to capturing temporal, sectoral and geographic mobility in one tool, with flexibility built in such that the tool can be pointed at an institution, a country, a region or a set thereof.

Following this introduction and literature review, we next discuss the questions and themes that arose in conversation with experts in researcher mobility in the university setting. Thereafter, we explain the data and methodology used, and then the results. We then discuss the various findings and their implications. We conclude by considering the benefits and limitations of this model as it exists to date and suggest future work.

### Consulting practitioners and experts connected to researcher mobility

As our intent with this study was to develop a researcher mobility model in line with the needs of practitioners, we took the decision to speak with employees at research institutions who were interested in the topic. We therefore sought the opinions and needs from those in university settings with an interest in researcher mobility.

In total, we conducted interviews with 35 employees from 12 institutions. We sought an international perspective and so the interviewees were based in the UK, Brazil, Australia, Columbia, Ecuador, Mexico and Sweden. The interviewees were working either as practitioners (bibliometricians, ranking specialists, senior researchers, senior research administrators, consultants) or institutional leaders (Pro Vice Chancellors, Deans, and Directors of Research, Innovation and Internationalisation). In these semi-structured interviews, conducted by the authors with support from colleagues with expertise in interviews, we discussed how researcher mobility impacts research institutions and exactly whose mobility are institutions interested in. All interviewees were selected for their expertise and interest on the topic at hand and were known contacts of the authors of the paper and their extended network.

The interviews highlighted the close link between researcher mobility, internationalisation strategies, staff policies, alumni relations and university success in research, an institution’s capacity to appeal to new postgraduate students, recruit new research or academic staff and attract external funding. All of the conversations moved between these topics. Below, we draw out some of the common themes that appeared throughout the interviews including the perceived benefits and outcomes of researcher mobility and the need for tracking. Per the interviewees’ requests, quotes are provided anonymously.

### Definitions and forms of researcher mobility

The interviewees reflected on the mobility of researchers in a variety of forms. Movements may be short-term, e.g., a few days or weeks spent in another university, or so-called ‘sabbatical mobility’ where dedicated funding facilitates visits to other universities. Of course, mobility may also be of a more permanent nature, e.g., researchers leaving the institution for another one. Interviewees also spoke of non-traditional mobility: one gave the example of facilitating ‘digital mobility’ where universities offer access to their resources (data, equipment) to former staff members. The provenance of researcher movements varies too: visiting opportunities for researchers, for example, are often developed from initial digital connections or through conference contacts by the researchers themselves.

### Mobility is tied to the broader sense of an institution’s level of internationality

The interviewees all considered researcher mobility to be deeply connected with and/or a driver of their institution’s internationality: that is, the extent to which institutions partake in research as a global enterprise.

The internationality of a university is important to its perceived ‘health’ and ability to conduct quality and impactful research. Interviewees highlighted the ways in which the internationality of their postgraduate programs or faculty are used in program evaluation and can help secure funding, and feeds into university rankings. Indeed, funding was mentioned by several interviewees as one of the drivers for the focus on researchers’ mobility. Countries in Latin America, for example, look abroad for funding: international funding opportunities often require international collaborations, and mobility is seen as “a bridge which enables a university to participate in large international projects, to receive external funding and to have articles with good impact”.

Universities adopt different mechanisms to boost researchers’ mobility. One interviewee noted rewarding researchers who published at least three outputs with an external partner following an outgoing short-term mobility as an example. Another interviewee mentioned offering incentives in the form of short-term teaching or research contracts to incoming visiting-researchers.

### Forging and cementing lasting relationships between institutions

Interviewees claimed that researcher mobility, even short term, can help create and cement long term relationships between universities, and those relationships can drive future research through continuing collaborations between the mobile researchers.

Researchers’ interactions were positioned as the cornerstone for building research partnerships by some interviewees. As well as leading to long term relationships, shorter visits to other institutions can become the basis for career changes and even institutional partnerships. Interviewees also reflected on the human element at play: “…what drives new research developments is people meeting people and identifying the ways in which they can collaborate, understanding that … there is a person that they’re able to work with.”

### Mobility encourages an international cohort of researchers with varied perspectives

The interviewees mentioned various benefits of researcher mobility on their institution’s networks and culture, the result of which is research with wider consideration of different perspectives. Collaborations between researchers (brought out by their mobility) tend to recur and can be beneficial to all parties in terms of shared knowledge, and access to resources and facilities.

### Overheads associated with internationality and mobility

The interviewees also reflected on the institutional overheads that help drive internationality. Short term positions and visits require investment from support departments, e.g., an International Office involved in promotional activities, arranging visas or relocation plans. As one interviewee explained, “it’s a constant juggling act for … universities with limited funding in terms of encouraging people to be active internationally”.

### Tracking researcher mobility is important to universities

The interviewees reported an acute need for an evidence-based approach to researchers’ mobility. In recognition of the above discussion points, the key drivers are to support strategic decision-making, to capture return on investment and to benchmark a university’s performance and position relative to others. Where universities have supported–especially financially–collaboration or exchange initiatives among universities, one of the measures of success is the movement of participating researchers between the universities.

There was also a desire to understand a university’s position in the research landscape in the context of researcher mobility and career paths: “Do people come to us in their early careers, and we are a stepping-stone, or do they come to us at the end of their careers?”. There were requests to see data on different types of mobility–national / international, newly recruited researchers / long-time collaborators–as well as researchers who are not mobile. Other interviewees were interested in a broader understanding of differences in movement patterns by discipline and career age across the global research enterprise.

On a practical level, several interviewees desired to be able to contact ex-employees where their expertise was thought to be of use to a project, particularly in the absence of the institutional memory or corporate systems.

The interviewees also recognized the risks of not monitoring mobility: “We don’t know what we don’t know, and sometimes it is a revelation to see that there were connections with certain parts of the world”.

Alongside mobility patterns, interviewees consistently mentioned the need to then understand productivity and impact of their researchers. Impact was a recurring theme, including both research and societal impact. For any interviewee who contributes to reporting research impact in order to secure funding, the need to factor that into researcher mobility tracking was made clear–especially given how well-reported it is that research which occurs through as a result of international collaboration, is in general, relatively more highly cited than otherwise.

### Summary and resulting impact on the model

These interviews were conducted after the conceptualization of this project, but prior to the development of all aspects of the model, allowing us to incorporate elements of the feedback into the research project design. In the particular instance of the model being proposed in this paper, some interviewees also felt that some form of validation of the data would be a key aspect of the project.

Input on the definition of an “active researcher” was also received. As there are differences in the publishing frequency between scientific fields, one proposition was to use a field-weighted active researcher definition.

In summary, as one interviewee put it: “Ultimately, the question is: how can this [researcher mobility model] help us improve the quality of the research at our university, or the researchers who will be recruited?”.

The interviews confirmed the interest in tools to study researcher mobility. While no single interview substantially changed the approach we took to the model, the compiled results gave us the trends and signals of important elements that we ensured were then built in.

## Materials and methods

### Data collection

As the source of this data, we choose the publications indexed in Scopus, as this is one of the largest curated abstract and citation databases of peer-reviewed literature. Scopus has been previously validated in terms of its use as a bibliometric data source for large-scale analyses in research assessments, research landscape studies, science policy evaluations, and university rankings [11].

For our case study, we selected all the publications from researchers who were affiliated at some point in their career with any (or multiple) Swedish institution in the period 1970–2021, resulting in 987,041 distinct documents (the documents’ metadata was used to identify author affiliation data for each publication). From these publications, we identified 278,153 unique authors. We then expanded the dataset to include all publications (no matter the affiliation) dated between 1970 and 2021 for these authors, resulting in 5,103,910 documents published by these researchers (with Swedish institutions or not). For authors with multiple affiliations listed on a publication, we consider all the affiliated institutions, and full counting was used to attribute credit to authors for mobility.

A necessary step prior to the construction of researchers’ mobility paths is the consolidation of the authors’ profiles which is ensured in Scopus by the Scopus Author ID (algorithms group publications of the same author by finding some similarity among them or directly assign publications to the individual who wrote them) and re-enforced by the author disambiguation services available (authors can consolidate their profile and collect together publications indexed in Scopus if these were allocated to more than one ID).

A significant complexity when dealing with publication data is the level of inconsistency associated with the affiliation names. To prevent recording false-positive cases of institutional mobility (when researchers moved across different sub-units / departments from the same institution) we used the SciVal institution IDs / names. SciVal is a research assessment tool, also provided by Elsevier, which allows access to research performance of research institutions and countries through analysis of Scopus data. The SciVal hierarchies consolidate Scopus affiliations under the same umbrella identifier when they are part of the same institution.

We developed a longitudinal perspective of researcher mobility at Swedish academic institutions through the period 1992–2021, further dividing this into three periods of 10 years.

If the authors didn’t publish in a certain year, then the most recent publication (versus that respective year) is informing authors’ affiliations.

The specific country and years of study applied here are for demonstration and the purposes of exploring our case study research questions; in other case studies different parameters may be selected. The researcher mobility model has the flexibility of changing both the reference year at the end of a timespan (any year from 1980 onwards) and the length of that period of time (i.e., 3, 5, 7, 10 years, etc.). Alongside the option of choosing any SciVal Institution ID and any country, these flexible parameters make the model flexible in identifying mobility patterns for researchers affiliated with specific institutions.

### Parameters

The data analysis solution was developed as a set of four nested PySpark notebooks [34] on Elsevier’s cloud-based computational platform powered by Databricks. There are several parameters (embedded as widgets in these notebooks), which makes this solution easy to adapt, re-purpose and run again at the next iterations of this analysis: country, target institution, last year of the analysis, timespan of the analysis, number of years used to define active researcher.

### Defining active researchers

Having identified all researchers who were affiliated to Swedish universities, we then identified the active researchers, and we considered publications as a signal of that activity. We used the pattern of publication as a demonstration of researcher’s activity and, complimentary, a sequence of consecutive years in which the author didn’t publish as a signal of becoming inactive; we based these on the Scopus-indexed publications as our data source. Below we detail what patterns we considered and used.

A lot of effort was invested in finding a definition of what constitutes an active researcher. As a starting point, we used a definition that Elsevier has used in previous approaches to study researcher mobility [35]. It defines an active researcher as someone who has published within the last five years and has on average at least one publication every third year since the first publication. However, the average requirement was found to be problematic in this case, as it made it difficult for researchers who had not published during a longer period to become active again; something that we wanted our model to capture. If a researcher publishes a certain year, he or she is probably to be considered active that year.

Therefore, a simpler definition was adopted: a researcher is considered active if he or she has at least one Scopus publication during the last X years. To give an example, if X is 4 and the last year included in the study is 2021, the researcher must have at least one publication during the years 2018–2021. As there are significant differences between the productivity rate across different scientific fields [36], we decided to make X a parameter that can be changed by the user of the model.

However, for the case study presented in this article, we had to derive a value for X. The selection of the value is basically a compromise between missing researchers who do not publish very frequently and including researchers who will never publish again. Based on data for 28 Swedish universities with substantial research output, we studied the results where the value of X varied from 1 to 8. The pattern was similar for all universities and when X increased from 1 to 2, the number of researchers included increased by 25–35 percent. In the other end, when the value of X increased from 7 to 8, the increase in the number of researchers was approximately 5 percent for all universities. As it is likely that very few researchers only publish every 8 years [37], the increase from year 7 to 8 relates mainly to the inclusion of researchers who do not publish anymore. In other words, the data shows that approximately 5 percent of the researchers in Sweden retire every year.

For our study, we gave X a value of 4, which corresponds to a situation when the number of researchers added to the cohort is higher than 10 percent, which means that active researchers who seldom publish constitute the majority. Again, the model is built with multiple use-cases in mind: as such, the value of X can be changed, informed by the characteristics of the cohort of researchers at hand, e.g., the trends in publication frequency if a certain discipline is selected.

### Indicators

A number of indicators are used in this study.

Career age is derived from the number of years between the researcher’s first publication and the most recent year of analysis. Researchers are then grouped into the following 3 categories: early career researchers (ECRs, 0–9 years since time of first publication), mid-career researchers (MCRs, 10–19 years since time of first publication) and late-career researchers (LCRs, 20 years or more since time of first publication).

Temporal mobility is defined using a taxonomy which was developed based on the affiliations linked to an author as reported on publications over the years. We use the publication(s) per calendar year to define where the researcher is. Firstly, we identify researchers’ status versus the Swedish institution selected for analysis: based on the yearly affiliation data (from all the publications in a selected or target year), researchers will be coded as: ***1*** if they were uniquely affiliated with the institution selected for analysis (the target institution); ***2*** if they were affiliated with the target institution alongside other institution(s) (either by having multiple affiliations on one publication or by having different affiliations on several publications); ***3*** if in that year they were not affiliated with the target institution, but only with other institutions; ***0*** if the researcher is not active. If an author doesn’t publish in one year their “position” remains the same as in the most recent year with a publication. For an example of how affiliation data is coded see Table 1.

**Table 1:** From affiliation data to mobility codes.

Mobility code	Affiliations
1, Target institution only	Target institution
2, Target institution and Others	Target institution and Other institutions
3, Other institutions only	Other institution

Secondly, the combinations of these codes are used to label researchers’ temporal mobility as follows (for details see Table 2).

- Not yet mobile: researchers were affiliated only with the selected institution throughout the timespan of the analysis, so for a given timespan, the years of the analysis are coded only as ***1*** (e.g., 1111 or 11 or 1).
- Visitor elsewhere: researchers were affiliated with the selected institution or with the selected institution and other institutions (in the same year), so only combinations of years coded ***1*** and ***2*** are present for the timespan of the analysis (e.g., 12221 or 121212).
- Stay elsewhere: in the most recent year of the analysis’ timespan researchers published with the selected institution or the selected institution alongside other institutions, while in previous years researchers had sometimes published exclusively with other institutions, therefore combinations of codes ***1*** or ***2*** and ***3*** (with 3 not in the most recent publishing year of the analysis’ timespan). For example, 121231 or 3212, etc., but not 32123 or 213.
- Visitor at institution: researchers were affiliated with the selected institution as well as other institutions, so only combinations of codes ***2*** and ***3*** (e.g., 232, 323 or 2232).
- Stay at institution: in the most recent year of the analysis’ timespan, the researchers published only with other institutions or the selected institution alongside other institutions, while in the previous years, the researchers had sometimes published exclusively with the selected institution, therefore combinations of codes ***2*** or ***3*** and ***1*** (with 1 not in the most recent publishing year of the analysis’ timespan). For example, 12123 or 3212, etc., but not 321231 or 2131.

**Table 2:** From mobility codes to temporal mobility definitions.

Temporal mobility	Last location	Previous locations
Not yet mobile	1, Target institution only	1, Target institution only
Visitor elsewhere	1, Target institution only	2, Target institution and Others
Stay elsewhere	1, Target institution only	3, Other institutions only
Stay elsewhere	1, Target institution only	2, Target institution and Others or 3, Other institutions only
Visitor elsewhere	2, Target institution and Others	2, Target institution and Others
Stay at target institution	2, Target institution and Others	1, Target institution only
Visitor at target institution	2, Target institution and Others	3, Other institutions only
Stay elsewhere	2, Target institution and Others	1, Target institution only or 3, Other institutions only
Stay at target institution	3, Other institutions only	1, Target institution only
Visitor at target institution	3, Other institutions only	2, Target institution and Others

Geographic mobility: using Sweden as the reference point, we use the model to explore movement in and out of the country. Researchers could have a “National mobility” (researchers affiliated only with Swedish institutions throughout the timespan of the analysis), an “International mobility” (researchers affiliated only with institutions from other countries than Sweden), or a “National and International mobility” (researchers affiliated with institutions from Sweden and other countries).

Sectoral mobility: using the higher education sector as the reference point, we use the model to explore movement across sectors, e.g., government or industry. Researchers could have an “Only academic sector mobility” or an “Academic and other sectors mobility” or an “Other sectors only mobility.”

Mobility flow: using researchers’ first, last and intermediary affiliations throughout a defined time period, alongside their active status in the years at the start and the end of the timespan, they could be categorised as follows (for details see Table 3):

- Perennials: researchers were affiliated with the selected institution or with the selected target institution and other institutions in the first and the last year of the analysis, so codes ***1*** or ***2*** are present at both the start and the end of the timespan. These are researchers who started at the target institution and remained throughout the time period of the analysis or returned there by the end of the timespan, being either uniquely affiliated (publishing only with that institution) or co-affiliated (affiliated with that institution and other institutions). We use this term, perennials, to reflect the ongoing connection–if not continuous–between the researcher and the selected institution.
- Inflow: researchers were affiliated with the selected institution (exclusively or not) in one of the years of the analysis timespan, so combinations of the codes ***1*** and ***2*** alongside ***3*** are present for the timespan of the analysis. These are researchers who were associated (published) with the target institution at some point throughout the period of the analysis (being either uniquely affiliated or co-affiliated).
- Outflow: the other situations, in which researchers who were affiliated with the selected institution or with the selected institution and other institutions in one of the years of the analysis, are either inactive or not uniquely affiliated with the target institution at end of the timespan. Therefore, combinations of the codes ***1***, ***2*** and ***3*** are present for the timespan of the analysis, with 1 not appearing in the most recent year of publication versus the end of the time period. These are researchers who, after publishing with the target institution at some point throughout the timespan of the analysis, end up not being uniquely affiliated with the target institution in the most recent year of publication or affiliated with it more than 4 years previously (due to being inactive at the end of the time period).

**Table 3:** From mobility codes to mobility flow definitions.

Mobility flow	First year of the period	Intermediate locations	Last year of the period	Definition
Perennials	1, Target institution only		1, Target institution only	Researchers with an ongoing connection with the target institution
	2, Target institution and Others		2, Target institution and Others	
Inflow	Not active	1, Target institution only or 2, Target institution and Others	Not active	Researchers who became affiliated with the target institution at some point throughout the period of the analysis
	Not active		1, Target institution only	
	Not active		2, Target institution and Others	
	Not active	1, Target institution only or 2, Target institution and Others	3, Other institutions only	
	2, Target institution and Others		1, Target institution only	
	3, Other institutions only		1, Target institution only or 2, Target institution and Others	
Outflow	1, Target institution only		Not active	Researchers who are affiliated to in the target institution but who become inactive or who are not uniquely affiliated with the target institution at the end of the study period
	2, Target institution and Others		Not active	
	3, Other institutions only	1, Target institution only or 2, Target institution and Others	Not active	
	1, Target institution only		2, Target institution and Others or 3, Other institutions only	
	2, Target institution and Others		3, Other institutions only	
	3, Other institutions only	1, Target institution only or 2, Target institution and Others	3, Other institutions only	

## Results

### Sweden’s profile amongst other countries

#### Sweden’s research—position in the world

Sweden is amongst the most prominent countries for research in the world, ranked 19^th^ in the most recent decade based on volume of scholarly output and 7^th^ using the Field Weighted Citation Impact (FWCI).

**Fig 1:**
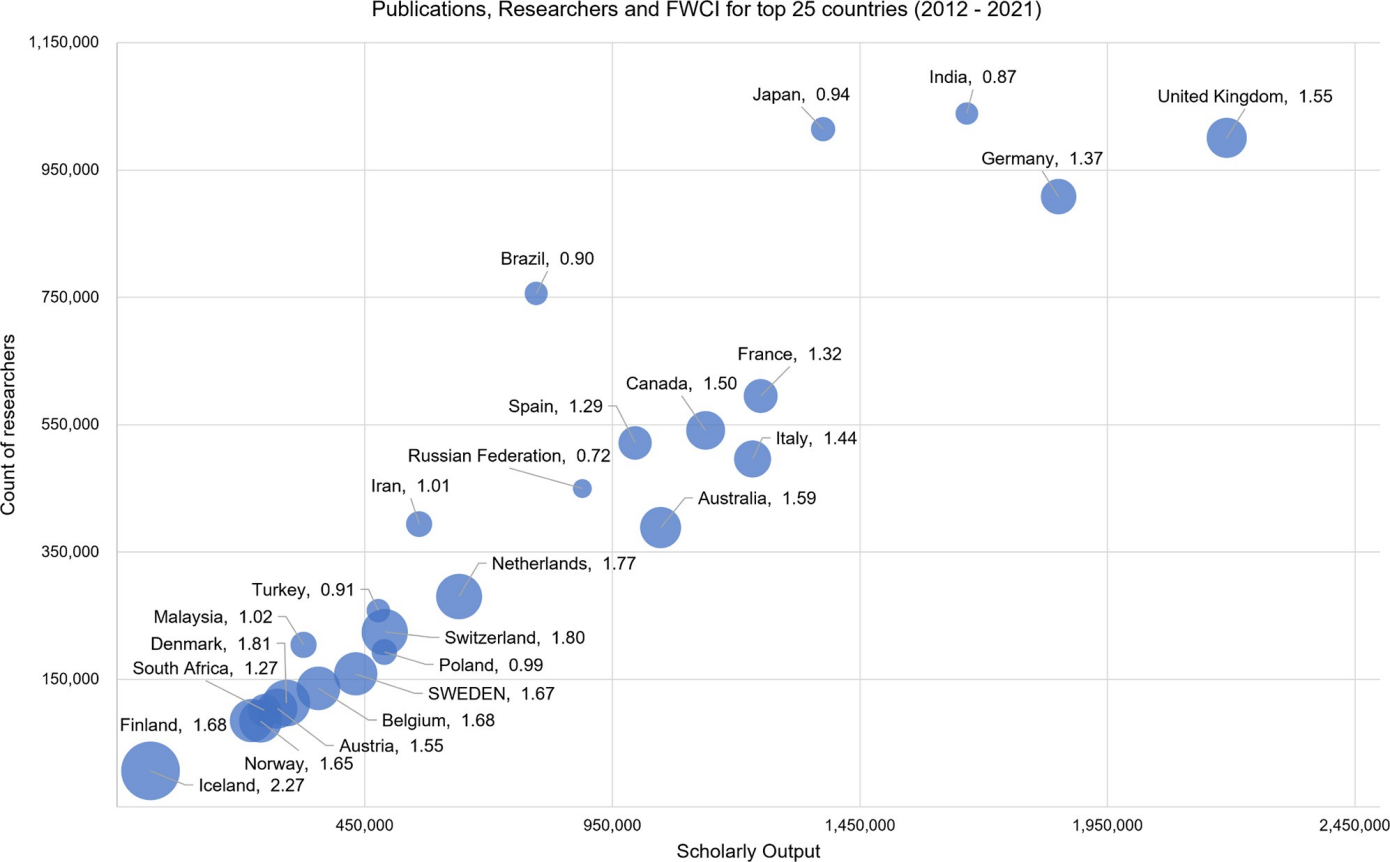
Sweden’s position amongst other countries, based on several indicators, 2012–2021. The indicators are: scholarly output (defined as the count of articles, reviews, books, books chapters and conference papers per year), Field Weighted Citation Impact (FWCI) and count of unique researchers. The size of node indicates the relative Field Weighted Citation Impact. Note: As outliers, USA and China’s values are not presented on this figure.

We used the researcher mobility model to identify the number of researchers who were affiliated throughout 1992–2021 with the top 25 countries (based on the scholarly output) to which we also added the Nordic countries. Based on the number of researchers, Sweden retained a top 20 position in the world in 2012–2021 (see S1 Table). In line with other reports [38], the data highlights the particularly high growth in researcher populations in several countries including China, India and Brazil. Alongside Sweden, the other Nordic countries had a similar or higher percentage increase in the number of researchers albeit from a smaller basis. Fig 1 shows the position of Sweden’s research in the world based on the three indicators.

#### Sweden’s research—position amongst the competitors

**Fig 2:**
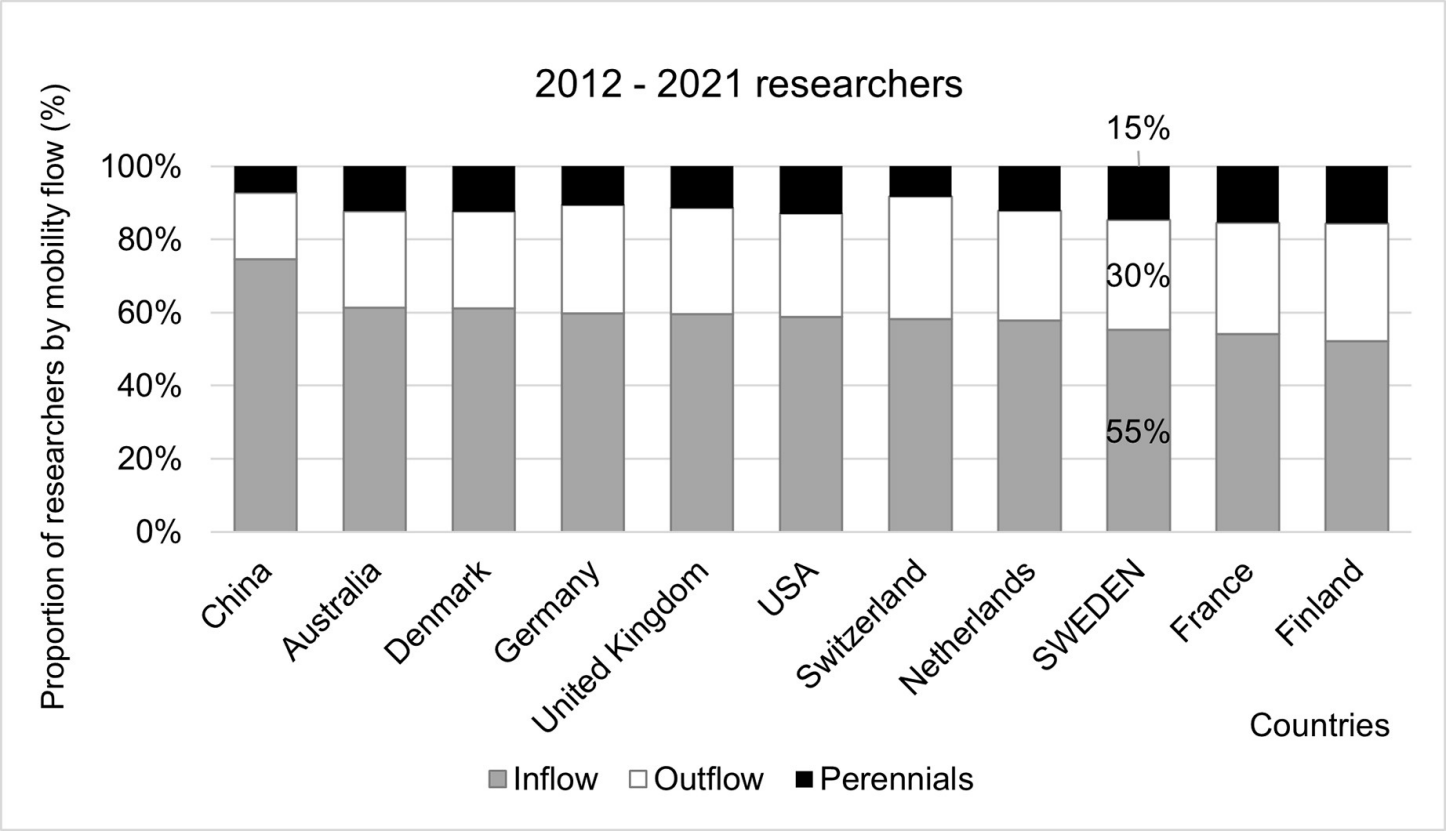
Proportions of researchers affiliated with Sweden and comparator countries by mobility type. Data sorted by proportion of Inflow researchers in 2012–2021. Affiliation on at least one publication during the period 2012–2021.

Using the mobility model and the mobility flow labels, we identify that the typical country profile has a high proportion of Inflow (around 60 percent), about a quarter of researchers categorised as Outflow and a small cohort of Perennials. In Fig 2 we focused on countries that frequently collaborate with Sweden (in the top 20 list of co-publications with Sweden) and which represent different regions and profiles. Among these comparator countries, Sweden has among the highest proportion of Perennials (15 percent, from only 10 percent in 1992–2001) and lowest percentage of Inflow researchers (55 percent), excepting France and Finland. China, notably, has a slightly different profile than the other countries, with more than 75 percent researchers identified as Inflow.

#### High intake of early career researchers associated with overall growth

**Fig 3:**
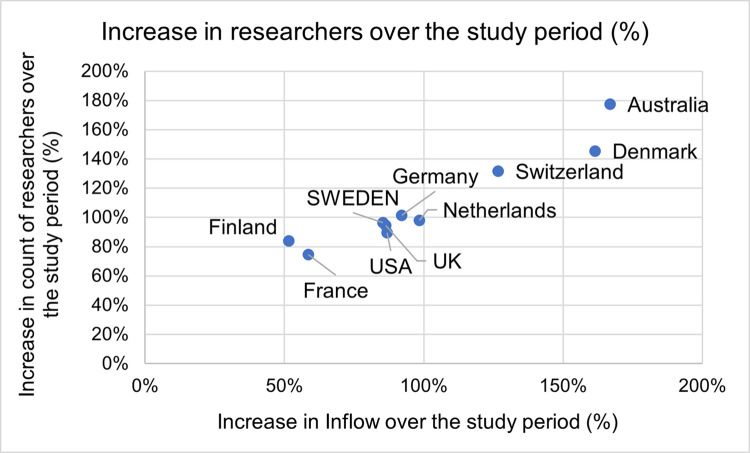
Increase in total count of researchers and increase in the category of inflow researchers. Data for Sweden and selected countries between 1992–2001 and 2012–2021. Note: As an outlier, China’s values (1100 percent increase in Inflow and 1200 percent increase in count of researchers over the study period) are not presented on this chart.

High proportions of researchers categorized as Inflow are positively correlated with the increase in the number of researchers (Fig 3). Since early career researchers (ECRs, 0–9 years since time of first publication) make up a high proportion of the Inflow cohort across all countries (84–98 percent) we can conclude that this segment of researchers represents one of the main drivers for growth in the number of researchers.

#### Mobility flow and career age in Sweden

**Fig 4:**
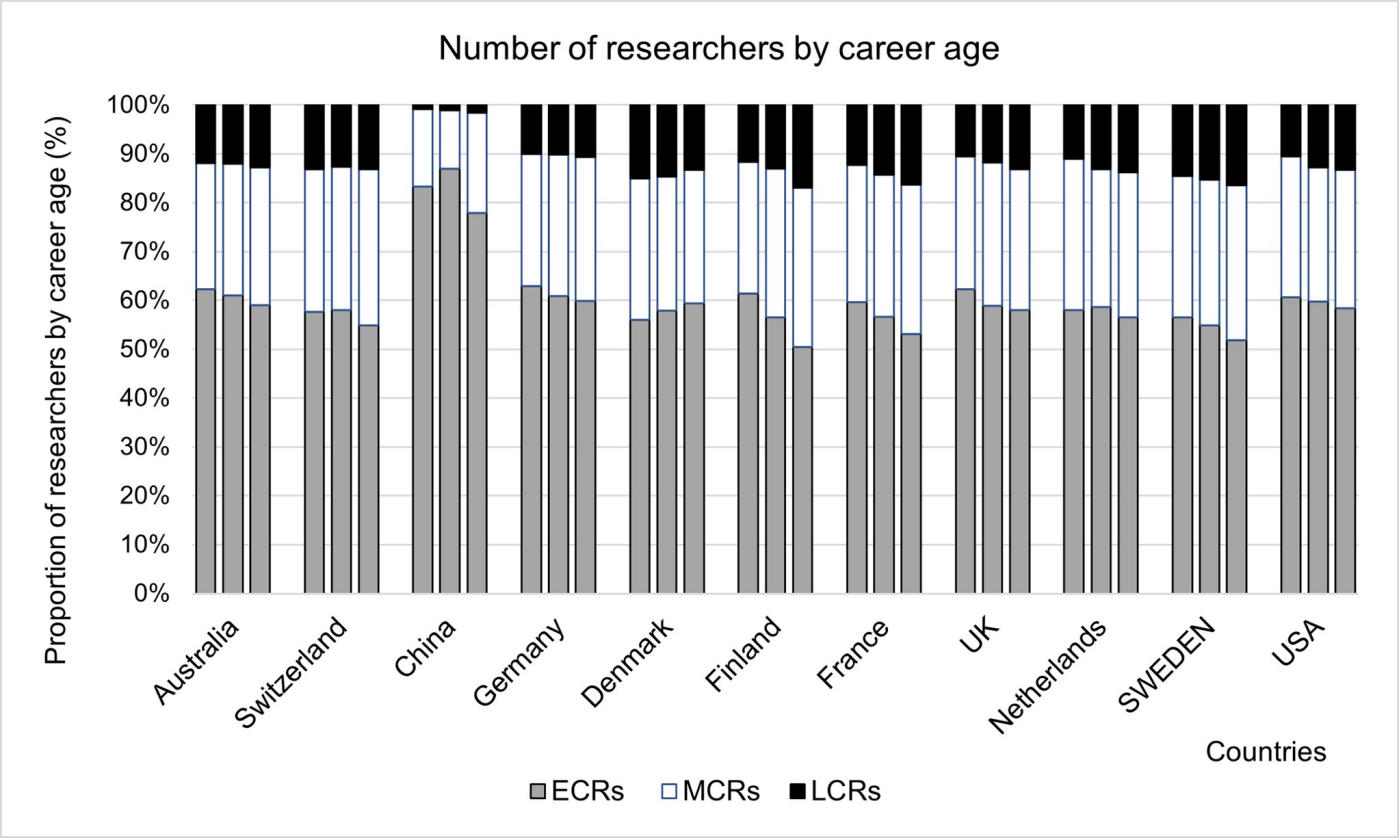
Proportions of researchers by career age for Sweden and other countries, 2012–2021. Note: The bars in each group represent the decades 1992–2001, 2002–2011, 2012–2021 arranged left to right.

ECRs represent over half of the total number of researchers affiliated with a Swedish institution in the most recent decade and there is a similar distribution for the comparator countries, bar China (Fig 4). However, their proportion decreased slightly throughout 1992–2021. The number of mid-career researchers (MCRs, 10–19 years since time of first publication) more than doubled between 1992 and 2021 and their proportion increased to represent now a third of all researchers; the percentage of late career researchers (LCRs, 20 years or more since time of first publication) increased only slightly and represents around 15 percent of the total count. China is notable for its particularly high proportion of ECRs and very low proportion of LCRs, with both segments showing the same trend as for the other countries (decreasing proportion of ECRs and increasing proportion of MCRs / LCRs).

**Fig 5:**
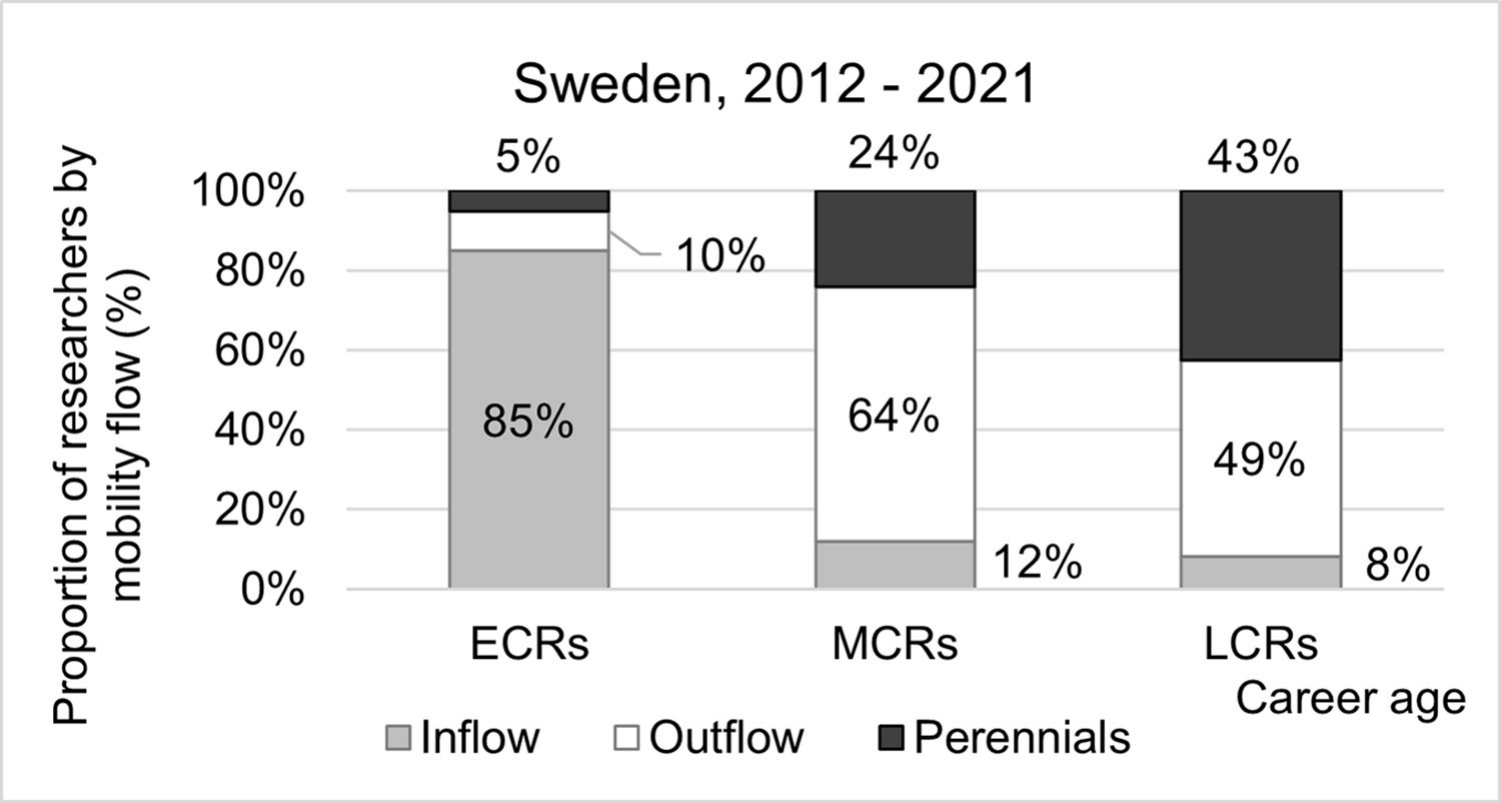
Proportions of early, mid and late career researchers in Sweden by mobility flow, 2012–2021.

The distribution of researchers in Sweden by type of mobility and career age shows that MCRs and LCRs account for most of the Perennials, in almost equal parts, while in the Outflow segment the MCRs are predominant (Fig 5). The proportion of LCRs classified as Perennials increased throughout 1992–2021 from 26 to 38 percent, driven by a reduced proportion of Outflow. The increase in proportion of Perennials across the career ages suggests international mobility decreases throughout a researcher’s career.

#### Swedish higher education sector

For our study, we are interested in the Swedish research landscape. By necessity, the analysis of the Swedish higher education sector focused on 30 higher education institutions (HEIs) with a SciVal profile. According to “An Overview of Swedish Higher Education and Research 2022” report there are three types of HEIs: universities, university colleges and research institutes [39]. We focus our analysis only on the first two categories which are associated with significant numbers of researchers.

Using the researcher mobility model, we calculated the number of researchers affiliated with Swedish HEIs, which increased throughout 1992–2021, though at different rates across the various institutions.

**Fig 6:**
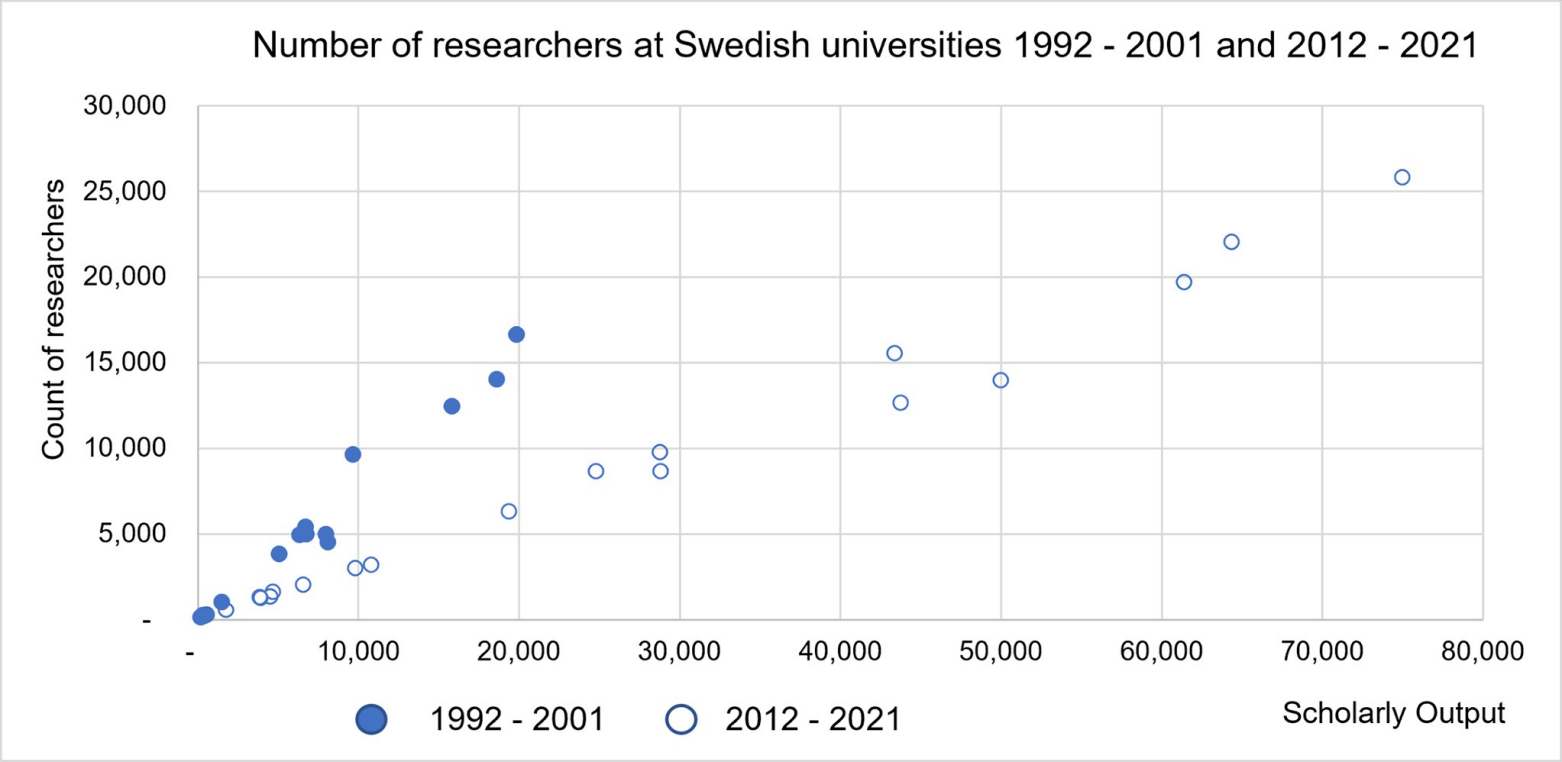
Number of researchers at Swedish universities, 1992–2001 and 2012–2021.

The Swedish universities vary widely in terms of the number of researchers, between 500 and 26,000 in 2012–2021 (from between 300 and 17,000 researchers in 1992–2001), reflecting their different profiles, capacity, areas of specialism. In the most recent decade, a third of the 18 Swedish universities had 10,000 or more affiliated researchers and only one of the HEIs–the more specialized Stockholm School of Economics–had fewer than 1,000 researchers. Despite the different rates of growth (Fig 6), the composition of this part of the Swedish higher education sector is quite stable, with most of the universities retaining their position in a ranking based on the number of affiliated researchers, with Karolinska, Lund, Uppsala, Gothenburg, KTH and Stockholm university being the largest.

The university colleges also increased the number of researchers affiliated with them. In the most recent period, 2012–2021, two thirds of the university colleges had 500 or more affiliated researchers as shown in S2 Table, while two decades ago none of the 12 university colleges had more than 500 affiliated researchers.

In a striking difference with the universities group, the Swedish university colleges experienced growth in the number of affiliated researchers but also fluctuations in the internal ranking, based on the number of affiliated researchers. The only stable fixture is Jönköping University constantly in top of position, followed by Blekinge Institute of Technology. In the most recent decade, we see an emerging middle echelon, with the more niche, focus-specific Swedish Defence University and Swedish School of Sport and Health Studies at the low end of the scale (S3 Table).

### Researcher mobility at Swedish universities

Swedish universities have a reputable profile across the world [40], due to the quality of research conducted. This is reflected in the high level of scholarly output and their high research impact, with most of the 18 universities having an average FWCI higher than 1.50 in 2012–2021, at the level of top research countries (Karolinska Institutet: 2.11; Stockholm University: 1.99).

All universities demonstrated growth in the count of affiliated researchers throughout 1992–2021 (see S3 Table), though, as probably expected, the overall growth in this sector stems from the largest universities, with the top 10 universities accounting for 76 percent of the growth in Swedish researchers throughout 1992–2021. For example, KTH Royal Institute of Technology and Stockholm University had particularly high growth (180 and 133 percent, respectively).

The majority of the growth in the number of researchers came from the Inflow segment (51–69 percent). For 4 universities (Umeå, Swedish University of Agricultural Sciences, Mid Sweden University and Stockholm School of Economics), the growth in the number of researchers comes in almost equal parts from Inflow as well as Outflow. The analysis of the growth in the number of researchers based on the career age segmentation shows that the typical profile is 50 percent ECRs, 35 percent MCRs and 15 percent LCRs, with only 3 universities for which the growth is mostly from MCRs (Stockholm School of Economics, SUAS and Umeå).

### University profiles

**Fig 7:**
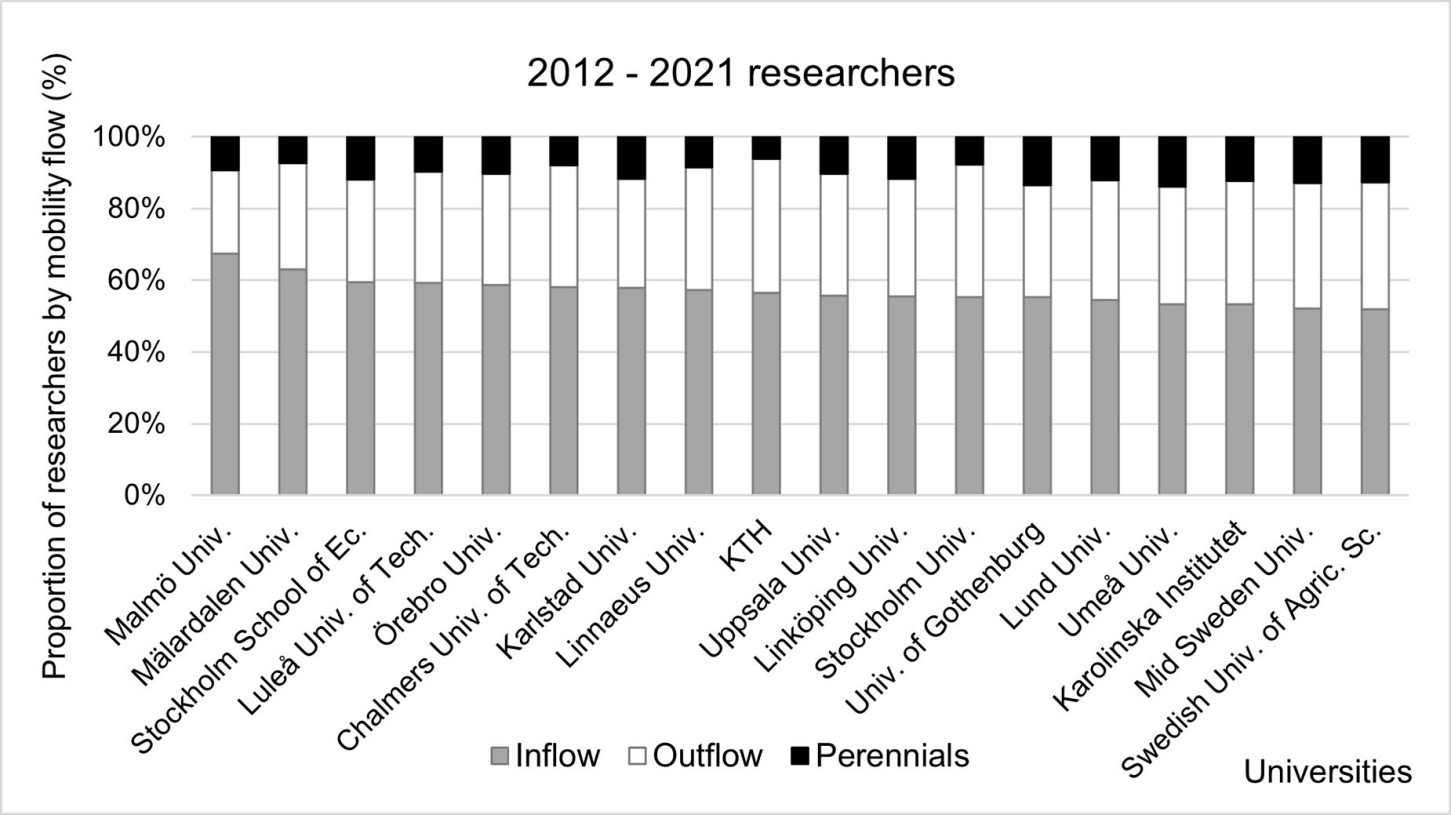
Proportions of researchers at Swedish universities by type of mobility flow. Data sorted by proportion of Inflow researchers in 2012–2021.

The typical Swedish university profile is 55 percent Inflow, 35 percent Outflow, 10 percent Perennials (Fig 7). Two universities stand out as having a higher proportion of Inflow researchers than the 55 percent country level: Malmö University and Mälardalen University. Both are relatively small (fewer than 1,600 researchers in 2012–2021) with a comparatively higher proportion of ECRs: 58 percent for Malmö University and 57 percent for Mälardalen University across 2012–2021.

We see the highest proportion of Outflow researchers amongst the largest Swedish universities (KTH, Stockholm University, Karolinska Institutet) and there could be a number of factors behind this: retiring academics; researchers leaving academia or moving to roles with less focus on publishing research; researchers moving to other institutions abroad; or external collaborators who return to their original institutions at the end of a short or long-term mobility.

**Fig 8:**
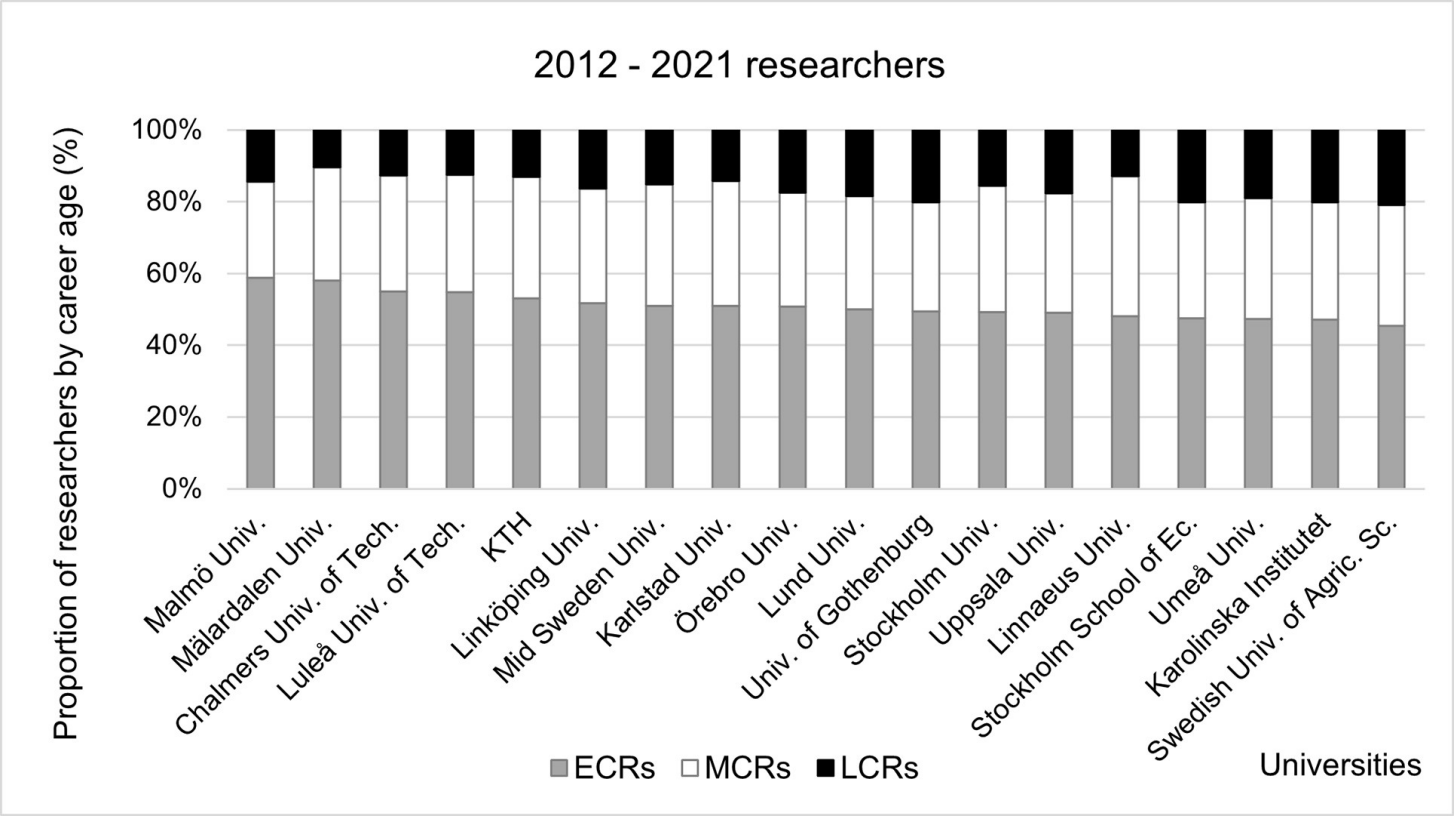
Proportions of researchers at Swedish universities by career age. Data sorted by proportion of Early-career researchers in 2012–2021.

The analysis of the distribution of researchers by career age (Fig 8) shows that smaller universities tend to have a higher proportion of ECRs compared to larger universities with the notable exception of KTH (53 percent). There is also a high proportion of Inflow and Perennials among LCRs in the smaller universities, so we can conclude that they are more adapted to attract ECRs and retain LCRs.

### Closer analysis for the six largest Swedish universities

Until now, we have explored the overarching mobility of researchers from a university and country perspective. But this high-level view does not yet demonstrate the transitory nature of research, nor the granular aspects of mobility, the direction of travel of the researchers, nor the different dimensions of mobility–be it across sector, country, etc. As the need for an understanding of these dynamics were called out by our interviewees, our model allows for a granular and multi-dimensional analysis of mobility. More specifically, the model enables us to drill down, at university level, to observe patterns in temporal mobility, sectoral mobility, and country movements. The model is also able to differentiate between researchers at different stages of their career. Finally, we can overlay these results with measures of productivity (publication counts) and research impact (citation counts).

In this section, we delve deeper into the researcher mobility for the 6 largest institutions by number of researchers, which had over 10,000 authors affiliated with them in the last decade. These are, in alphabetical order: University of Gothenburg, Karolinska Institutet, KTH Royal Institute of Technology, Lund University, Stockholm University, Uppsala University. For each of these universities, we have explored temporal, sectoral and geographic mobility, comparing the results by researcher career age.

For context, we apply the overarching view of mobility to all six universities before drilling down further: KTH and Stockholm University had the highest growth in the number of researchers amongst the 6 largest institutions between 1992–2021, in triple digits (174 and 134 percent growth, respectively) in comparison to 96 percent overall increase for Sweden. In comparison, the growth for the other 4 institutions was between 56 and 61 percent. The drivers of growth were different for these 2 universities: Outflow for KTH and Inflow for Stockholm University.

Examining in more detail, we see that KTH’s growth in number of researchers between 1992 and 2021 was relatively higher than that of Sweden as a whole across all career ages, with MCRs and LCRs segments having a particularly high increase (248 and 214 percent, respectively vs. 133 percent for ECRs). In 2012–2021, 70 percent of the MCRs were classified as Outflow, with three quarters of them having a stay or a visit at KTH throughout this period. The majority of this subgroup of researchers demonstrated International mobility (80 percent) and, using the sectoral mobility dimension, we found that around half of the group originated from the academic sector–the other half originated from academic *and* other sectors. This suggests that KTH attracts experienced researchers from both other countries and sectors for short and long periods of time. The wealth of knowledge transfer is reflected in the substantial increase in the number of outputs published by the university as a whole in 2012–2021 (double than the previous decade) and the improved FWCI (1.76). The growth in Outflow over the decades at KTH highlights that this university was more successful in attracting researchers, even if only for a few years of that decade, through shorter mobilities (like visits).

The majority of the growth in the number of researchers affiliated with Stockholm University between 1992–2021 came from the Inflow segment (143 percent increase vs. 134 for the university overall). Almost 80 percent of were ECRs and half of these already mobile. Notably, this is also the highest proportion of mobile ECRs in the Inflow segment across the 6 largest Swedish universities. Three quarters of these researchers were mobile within the decade at another university, split almost equally between Swedish and international institutions. As most of these researchers were affiliated (or co-affiliated) with Stockholm University on the first paper published at the start of their career, these findings suggest that this university is an incubator for ECRs that go on to high levels of mobility.

Examining at a more detailed level the different types of mobility, the variations in the profiles of institutions become apparent. For example, University of Gothenburg and Karolinska Institutet had the highest proportion of Perennials in 2012–2021 (14.7 percent and 13.3 percent, respectively, and a similar distribution by career age: 7 percent ECRs, 43 percent MCRs, 50 percent LCRs). However, using the temporal mobility indicator, we identify that a third of the Perennials at Karolinska Institutet published with Karolinska and at least one other institution, while for the University of Gothenburg these researchers represent only a quarter (the number of Not Mobile researchers grew at a higher rate than any other group, tripling to 1,500 throughout 1992–2021). It seems that Karolinska’s higher mobility rates in the Perennials segment is down to an increase in the short-term mobility abroad: 500 Visitors Elsewhere demonstrated international mobility for Karolinska in 2012–2021, double than throughout 1992–2001 compared to 200 Visitors Elsewhere demonstrated international mobility for University of Gothenburg, a 36 percent increase over the same period.

**Fig 9:**
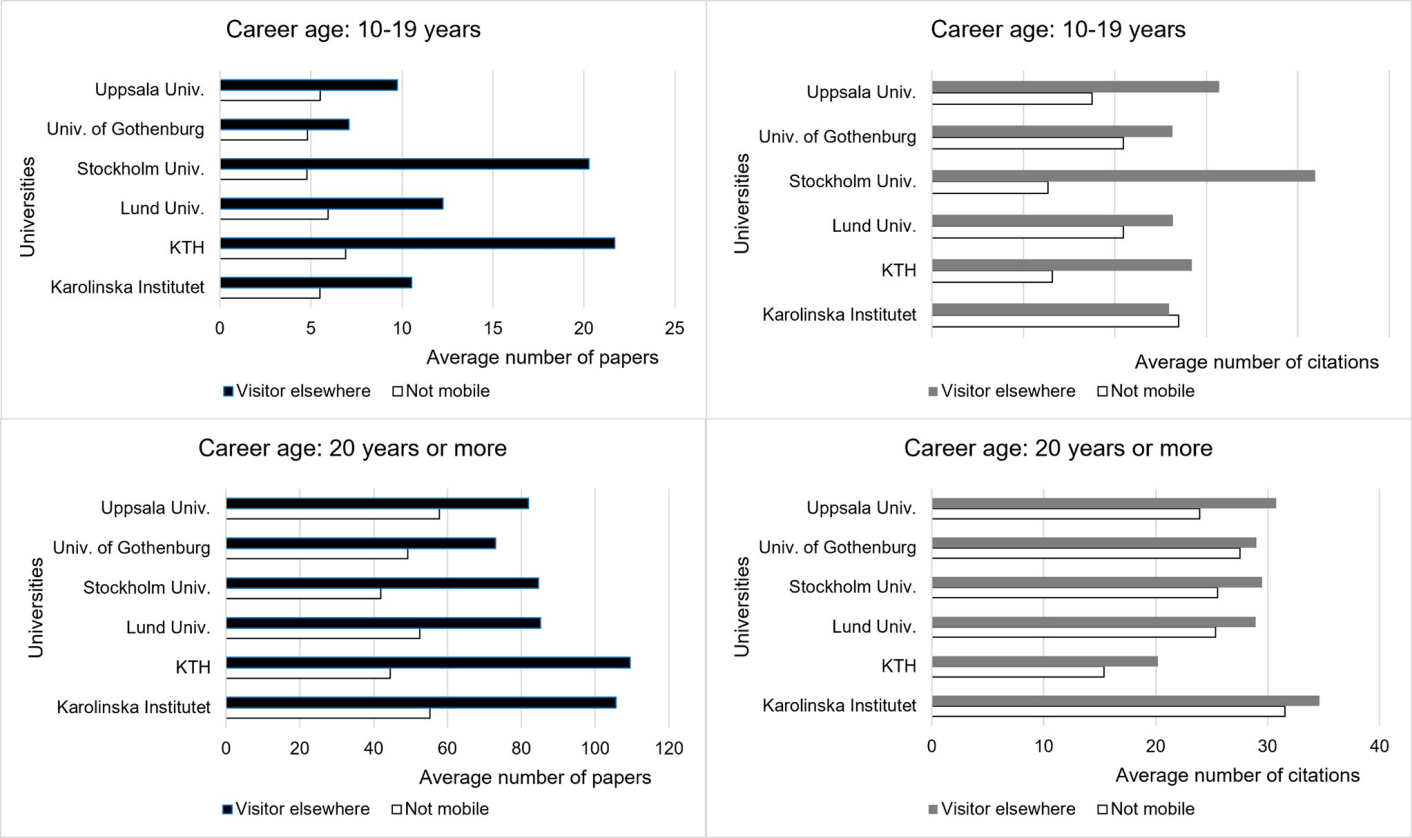
Comparison of the average number of papers and their citations. Data for Visiting and Not Mobile researchers across Sweden’s 6 largest universities, 2012–2021.

When comparing scholarly output for Visitor Elsewhere and Not Mobile across the 6 universities in the period 2012–2021 (Fig 9), we identify that Visiting researchers are consistently more productive by this measure. In line with other findings [1], we also see that research impact, measured by FWCI, is generally higher for Visiting researchers.

Most ECRs are, unsurprisingly, classified as Inflow as many of these will be starting their careers during the period of study; indeed, in the case of the 6 universities, ECRs represented between 78 and 82 percent of the Inflow category in 2012–2021. However, there are differences in the distribution of the ECRs by affiliated institution by the end of the 2012–2021: 75 percent of the inflow ECRs are still affiliated with Lund University, while this proportion decreased to 59 percent for Stockholm University. We also see differences in the proportion of ECRs who demonstrated International mobility: 56 percent for University of Gothenburg compared to 73 percent for Lund University. We also examined the countries that researchers moved to or were co-affiliated with (alongside the target institution), based on the location of the affiliated institution at the end of the decade. Among the countries of destination for the Inflow ECRs, we notice that a decreasing number of researchers were affiliated with USA (1 in 5 in 1992–2001; 1 in 10 in 2012–2021), UK or Japan, while others became more prominent (China, Germany, Netherlands, Spain) and new destinations emerged in 2012–2021 (Brazil, India, Iran).

There are some interesting fluctuations related to the countries of origin among the mid and late career Inflow researchers demonstrating any type of mobility of those we studied. In 2012–2021, LCRs were more likely to have been affiliated with an institution from USA, UK or one of the Nordic countries before their stay or visit with one of the 6 universities, while MCRs were more likely to have been affiliated with an institution from Germany, Spain, Switzerland, Canada, Netherlands or China, India, Brazil, Iran.

We see some differences between the profile of the Outflow researchers demonstrating mobility at one of the 6 universities: the proportion of MCRs is considerable higher than that of the LCRs (27–53 percentage points higher) in the case of a Stay than in the case of a Visit at the institution (2–26 percentage points). These results show that the MCRs are more likely to have a longer form of mobility with another institution than the LCRs, who seem to prefer shorter mobilities identified as visits (publishing with the target institution alongside others).

These larger universities in Sweden’s HE sector tend to see a high level of inter-university mobility: most of the Outflow researchers (66–79 percent) have International mobility (i.e., publish with both national and international institutions). The trends in the countries of destinations are more stable, although USA, UK, Italy, France, Japan and Belgium had a drop in the proportion of Outflow mobile researchers visiting / moving to these countries. In consideration of sectoral mobility, we also examined how researchers moved across sectors such as industry, medical and government: while at a lower rate than geographic mobility, we discovered that 37–40 percent of Outflow researchers were affiliated throughout a decade with organisations from academic as well as other sectors.

## Discussion

This study focused on Sweden, a top 20 country in the world based on a variety of indicators of research productivity and impact and the number of researchers. Sweden has some of the lowest percentages of Inflow researchers (55 percent, 1992–2001) and highest proportion of Perennials (15 percent, 1992–2001) among the comparator countries which collaborate frequently with Sweden.

The distribution of researchers in Sweden by type of mobility and career age shows that MCRs and LCRs account for most of the Perennials, in almost equal parts, while in the Outflow segment the MCRs are predominant. Smaller universities tend to have a higher proportion of ECRs compared to larger universities. We see the highest proportion of Outflow researchers amongst the largest Swedish universities.

Amongst the largest 6 Swedish universities we saw that there are different drivers of growth (Outflow for KTH, Inflow for Stockholm University) and different profiles (University of Gothenburg and Karolinska Institutet had the highest proportion of Perennials). The researchers affiliated with these institutions have different mobility patterns according to the career age. There are fewer researchers affiliated with USA, UK or Japan, new countries of destinations emerge (Brazil, India, Iran) and others become more prominent (China, Germany, Netherlands, Spain).

By testing the model on various aspects of the Swedish Higher Education sector and presenting the findings together, we aim to demonstrate how a researcher model that enables a flexible approach to the entities under consideration and the data that can be examined can build a comprehensive picture of researcher mobility.

In the prior literature, researcher mobility has been noted as an integral part of the research landscape and researcher careers [41]. Institutions [18], countries [6] and regions [13] all pay attention to the trends and patterns, in part to measure their ‘attractiveness’ and to identify knowledge flow. As such, we aim to contribute to the research literature with a study on Sweden’s researcher mobility.

Most of the growth in the number of researchers at Swedish universities came from the Inflow segment (51–69 percent), so one way in which the country could continue to increase the number of researchers at a similar level with its competitors would be to focus on increasing the Inflow, especially as this has dropped slightly as a proportion over time. Since ECRs make up a high proportion of the Inflow population, and Sweden is a relatively small country with an aging population [42] (as it is the case for most of the European ones), increasing the Inflow without constant efforts to invest in programs actively recruiting PhD students and postdocs from abroad will be problematic.

As the Inflow has implications for the balance of the Swedish researcher population, its institutions might want to consider how to attract more medium and late career researchers to address the balance. Increasing the Inflow through the MCRs and LCRs should be done in a targeted way, as the study showed that, for the 6 largest institutions, these researchers come from different markets (LCRs were more likely to have been affiliated with an institution from USA, UK or one of the Nordic countries, while MCRs were more likely to have been affiliated with an institution from Germany, Spain, Switzerland, Canada, Netherlands or China, India, Brazil, Iran). Moreover, the trends observed for 2012–2021 could shift in time, so a continuous review of the insights provided through a periodical run of the researcher mobility model to gather the relevant national and institutional data is advisable.

Sweden, like any other nation, might have an eye on both the number of Inflow and Outflow researchers given the international nature of science, and therefore should consider how to remain attractive to researchers: a healthy Inflow and Outflow ensures Sweden is part of the international scientific community, and continues to have a high impact, being in a good position to address the global challenges. The Swedish higher education sector is not a homogenous block, but, as it is the case in other countries, it is made up of very different constituent institutions in size, specialism, type of networks. We saw, for example, that smaller institutions are more adapted to attract ECRs and retain LCRs, that there are different drivers of growth amongst the 6 largest universities. Therefore, there is scope to analyse the distinct mobility patterns in each of the institutions to draw the specific conclusions and put in place customised, local measures which will build up in a successful story–that of the country retaining its top 20 research position.

The study showed that there is a relatively higher proportion of Perennials researchers in Sweden compared to other countries and throughout 1992–2021 the number and proportion of late career researchers classified as Perennials tripled–growing at a higher rate than the overall researcher population. It is important, therefore, to encourage short-term mobility of this segment as external collaborations are known to improve the volume and impact of research outputs.

### Further research

We see at least two different paths for future research. One path relates to using the model to study other aspects of researcher mobility than covered in this article. Another path relates to further development of the model.

Through this study, we have demonstrated that the model is amenable to a great deal of flexibility. However, the model is continuing work in progress, and there are further developments and tests we would like to conduct. In particular, we would like to test it on other countries and institutions from specific regions. We would also like to examine more deeply the categorization of different types of mobility to identify whether they are logical in all use cases, beyond a country level case study such as the one presented in this paper. The following iterations of this study will explore, for example, if identifying certain categories such as “Beginners” as a part of Inflow and “Not active” as part of Outflow could bring useful layers to future analysis; our focus in this study was on clarity and simplicity.

Future work might entail drawing in signals of economic and societal impact to overlay with the researcher mobility. There are also a variety of other dimensions which we want to add to this researcher mobility model, for example, researcher gender which could be inferred from publication metadata, as other studies have found differences when applying the gender lens [3]. We would also like to explore the relationship between researcher mobility and ‘stickability,’ namely what is the proportion of the net inflow of researchers who take-up permanent positions. This is an interesting idea which could be explored further by triangulating the data with external sources (ORCID, LinkedIn data, university repositories or HR systems data).

Even if it appears rather trivial to define different categories of researchers in terms of their mobility and activity, there is a need for further research to fine-tune logical and useful categories. One way to handle this is to make the category user defined. However, if such approaches are used too often, the model becomes difficult to use and comparisons of the results will become problematic.

Our vision is to provide a model available to actors in the higher education system, which allows them to carry out analyses in line with their interests and needs. Even though the current model has some flexibility, a lot of work remains to make a user-friendly interface. Also, there is a need to work with the presentation of data. For example, a Sankey chart could be useful to illustrate how the composition of researchers at a certain institution or country changes over a given period.

There are a few limitations to this study. An important limitation lies in that the model is reliant on publication metadata. Although this has largely been found to be sufficient for analyses of the kind we have undertaken, care must be taken with interpretation of the results [10]. To this end, we did conduct a short, supplementary comparison of Scopus metadata against researcher-managed career placements [43]. At this time, the model has been focused on Sweden. Further analyses might include other countries and regions, although we believe this study has demonstrated the flexibility and applicability. In particular, the model could evidence difference mobility patterns in different geodemographic contexts.

Secondly, the model uses Scopus’ curated author profiles to create researcher entities for analysis. In the case that one researcher is represented across multiple Scopus author profiles, this may mean that any institutional mobility is not or only partially captured. Because the Scopus author profiling system relies on various signals including author names, researcher populations that contain large amounts of extremely common names may experience a slightly lower mobility counts, and may consider implementing additional data cleaning before applying this approach.

The third limitation is the absence of the gender lens and we have plans to develop the model in this direction. Furthermore, the model–with adjustments to the code–is able to analyse the data over shorter time periods (3-year span instead of the decade approach, for example) and we intend to investigate in the next iterations of this study if changes in this parameter’s value would impact significantly on the current findings of our study. This could be included in further testing of the model, and would enable us to identify to what extent, in the recent years, mobility was affected by the COVID-19 pandemic. Although people were more mobile in the past, in the most recent years more online resources were made available due to travel restrictions. Thanks to technology, research collaborations could happen without the need for researchers to relocate; this was also illustrated by the work put in this project which involved, alongside the authors, other colleagues based in UK, Sweden, Netherlands and China.

Lastly, for the analyses to be most effective when considered by an institution or country, considered visualisation of the results would be worth deeper exploration.

## Conclusions

Building on the research literature on researcher mobility and the pre-existing approaches to measuring the phenomenon, this study aimed to enhance the ways in which researcher mobility was demonstrated: namely by introducing greater levels of flexibility in terms of levels of aggregation, a multi-dimensional approach to mobility in all its forms, and the data and indicators that can be overlayed to inform our understanding of other elements of research productivity and impact correlated with mobility.

With much experience of studying research mobility in a global capacity and by supplementing that knowledge with input from those in research institutions who are focused on the trends, we offer a tool which will serve in many different settings and can answer a variety of questions. Where different types of mobility are often dealt with in separate studies, our model brings together many dimensions. We believe that the refinements and advances presented here and, in particular the capacity that the researcher mobility model has for further expansion, contributes to a more comprehensive understanding of researcher mobility.

## Supporting information

S1 Table Sweden’s position amongst other countries, based on various indicators. (PDF)

S2 Table Number of researchers at the Swedish university colleges between 1992–2021. (PDF)

S3 Table Relative position between Swedish universities, based on various indicators. (PDF)
