# Supplementary material for: A bibliometric analysis using a newly developed model and a customizable research tool: A case study of researcher mobility in Sweden
Source: PLoS One. 2024 Dec 2;19(12):e0308147. doi: 10.1371/journal.pone.0308147 (PMC11611208; doi:10.1371/journal.pone.0308147)
Supplement: S1 Table — (PDF) [file pone.0308147.s001.pdf]

S1 Table. Sweden's position amongst other countries, based on various indicators.

Target country	Scholarly Output			Rank Scholarly Output			FWCI			Rank FWCI			Number of researchers			Increase researchers (%)		
	1996 - 2001	2002 - 2011	2012 - 2021	1996 - 2001	2002 - 2011	2012 - 2021	1996 - 2001	2002 - 2011	2012 - 2021	1996 - 2001	2002 - 2011	2012 - 2021	1992 - 2001	2002 - 2011	2012 - 2021	2011 v 2001	2021 v 2011	study period
China	274,829	2,183,457	5,839,495	*6*	*2*	*2*	0.44	0.66	1.00	*27*	*26*	*21*	383,841	1,669,183	5,010,194	*335%*	*200%*	*1205%*
United States of America	2,196,631	5,349,343	7,050,467	*1*	*1*	*1*	1.51	1.50	1.41	*6*	*9*	*14*	2,088,917	2,766,422	3,961,094	*32%*	*43%*	*90%*
India	142,213	550,331	1,665,665	*13*	*10*	*5*	0.55	0.74	0.87	*25*	*25*	*26*	146,138	339,449	1,038,901	*132%*	*206%*	*611%*
Japan	606,589	1,252,732	1,375,715	*2*	*5*	*6*	0.92	0.94	0.94	*18*	*19*	*23*	737,231	895,722	1,014,118	*21%*	*13%*	*38%*
United Kingdom	598,838	1,488,160	2,190,514	*3*	*3*	*3*	1.39	1.50	1.55	*8*	*9*	*10*	513,929	684,011	1,000,356	*33%*	*46%*	*95%*
Germany	514,833	1,279,188	1,851,038	*4*	*4*	*4*	1.24	1.36	1.37	*12*	*14*	*15*	450,920	615,960	908,208	*37%*	*47%*	*101%*
Brazil	78,288	363,648	796,087	*17*	*14*	*13*	0.74	0.83	0.90	*20*	*20*	*25*	94,199	310,502	756,369	*230%*	*144%*	*703%*
France	370,872	906,676	1,249,527	*5*	*6*	*7*	1.19	1.29	1.32	*15*	*16*	*16*	341,166	443,698	595,305	*30%*	*34%*	*74%*
Canada	264,350	761,048	1,138,262	*7*	*7*	*9*	1.39	1.48	1.50	*8*	*11*	*12*	248,174	351,841	541,114	*42%*	*54%*	*118%*
Spain	173,682	571,348	995,863	*11*	*9*	*11*	1.00	1.19	1.29	*17*	*17*	*17*	188,916	324,082	521,383	*72%*	*61%*	*176%*
Italy	255,066	716,843	1,233,134	*8*	*8*	*8*	1.12	1.31	1.44	*16*	*15*	*13*	252,473	337,875	496,356	*34%*	*47%*	*97%*
Russian Federation	205,704	387,349	889,338	*9*	*13*	*12*	0.49	0.56	0.72	*26*	*27*	*27*	193,132	217,929	449,873	*13%*	*106%*	*133%*
Iran	8,694	165,286	559,750	*25*	*20*	*15*	0.68	0.78	1.01	*22*	*22*	*20*	9,228	117,218	394,057	*1170%*	*236%*	*4170%*
Australia	173,695	540,022	1,047,465	*10*	*11*	*10*	1.23	1.43	1.59	*14*	*13*	*9*	140,114	224,365	388,591	*60%*	*73%*	*177%*
Netherlands	147,195	408,908	640,527	*12*	*12*	*14*	1.57	1.74	1.77	*2*	*4*	*4*	141,620	186,316	280,219	*32%*	*50%*	*98%*
Turkey	44,113	243,122	477,130	*22*	*18*	*18*	0.62	0.78	0.91	*23*	*22*	*24*	43,780	127,181	258,156	*191%*	*103%*	*490%*
Switzerland	104,303	291,155	490,132	*15*	*15*	*16*	1.64	1.79	1.80	*1*	*2*	*3*	96,917	141,513	224,405	*46%*	*59%*	*132%*
Malaysia	8,319	77,366	326,170	*26*	*26*	*21*	0.56	0.78	1.02	*24*	*22*	*19*	10,047	52,182	204,392	*419%*	*292%*	*1934%*
Poland	84,301	267,318	489,443	*16*	*16*	*17*	0.69	0.79	0.99	*21*	*21*	*22*	63,462	114,750	193,215	*81%*	*68%*	*204%*
SWEDEN	112,695	263,996	431,523	*14*	*17*	*19*	1.47	1.60	1.67	*7*	*6*	*7*	80,859	110,723	158,717	*37%*	*43%*	*96%*
Belgium	78,013	222,554	356,483	*18*	*19*	*20*	1.35	1.62	1.68	*10*	*5*	*5*	66,158	90,371	136,215	*37%*	*51%*	*106%*
Denmark	57,173	149,968	291,846	*20*	*22*	*22*	1.57	1.75	1.81	*2*	*3*	*2*	46,078	64,400	113,065	*40%*	*76%*	*145%*
Austria	52,802	159,592	273,499	*21*	*21*	*23*	1.24	1.45	1.55	*12*	*12*	*10*	42,910	68,493	104,419	*60%*	*52%*	*143%*
South Africa	31,130	97,370	247,363	*24*	*25*	*24*	0.86	1.12	1.27	*19*	*18*	*18*	29,885	46,829	101,686	*57%*	*117%*	*240%*
Finland	59,886	147,635	221,341	*19*	*23*	*26*	1.53	1.56	1.68	*4*	*7*	*5*	46,486	66,095	85,435	*42%*	*29%*	*84%*
Norway	39,906	121,770	239,469	*23*	*24*	*25*	1.35	1.56	1.65	*10*	*7*	*8*	30,526	48,493	84,407	*59%*	*74%*	*177%*
Iceland	2,196	8,159	17,439	*27*	*27*	*27*	1.53	1.94	2.27	*4*	*1*	*1*	1,843	3,585	6,433	*95%*	*79%*	*249%*

The indicators are: scholarly output (defined as the count of articles, reviews, books, books chapters and conference papers per year), Field Weighted Citation Impact (FWCI) and count of unique researchers. Data was sorted by number of researchers in 2012-2021. Data source: SciVal for scholarly outputs and FWCI, Researcher mobility tool for number of researchers.
