# Supplementary material for: A bibliometric analysis using a newly developed model and a customizable research tool: A case study of researcher mobility in Sweden
Source: PLoS One. 2024 Dec 2;19(12):e0308147. doi: 10.1371/journal.pone.0308147 (PMC11611208; doi:10.1371/journal.pone.0308147)
Supplement: S2 Table — (PDF) [file pone.0308147.s002.pdf]

S2 Table. Number of researchers at the Swedish university colleges between 1992-2021.

Swedish university college	Number of researchers affiliated			Size of the university			Decade increase		Rank			Improvement in rank	
	1992 - 2001	2002 - 2011	2012 - 2021	1992 - 2001	2002 - 2011	2012 - 2021	2011 v 2001	2021 v 2011	1992 - 2001	2002 - 2011	2012 - 2021	2011 v 2001	2021 v 2011
Jönköping University	132	537	1,103	under 500	500 - 999	1,000 - 4,999	307%	105%	*1*	*1*	*1*	0	0
Blekinge Institute of Technology	104	509	903	under 500	500 - 999	500 - 999	389%	77%	*3*	*2*	*2*	1	0
Halmstad University	110	356	768	under 500	under 500	500 - 999	224%	116%	*2*	*6*	*3*	-4	3
Dalarna University	84	291	738	under 500	under 500	500 - 999	246%	154%	*4*	*8*	*4*	-4	4
University of Borås	76	366	723	under 500	under 500	500 - 999	382%	98%	*7*	*5*	*5*	2	0
University of Gävle	58	345	693	under 500	under 500	500 - 999	495%	101%	*8*	*7*	*6*	1	1
Södertörn University	78	453	653	under 500	under 500	500 - 999	481%	44%	*6*	*3*	*7*	3	-4
University of Skövde	83	382	631	under 500	under 500	500 - 999	360%	65%	*5*	*4*	*8*	1	-4
University West	30	219	511	under 500	under 500	500 - 999	630%	133%	*11*	*9*	*9*	2	0
Kristianstad University	50	209	402	under 500	under 500	under 500	318%	92%	*9*	*10*	*10*	-1	0
Swedish Defence University	27	111	207	under 500	under 500	under 500	311%	86%	*12*	*11*	*11*	1	0
Swedish School of Sport and Health Sciences	36	108	200	under 500	under 500	under 500	200%	85%	*10*	*12*	*12*	-2	0
