# Supplementary material for: A bibliometric analysis using a newly developed model and a customizable research tool: A case study of researcher mobility in Sweden
Source: PLoS One. 2024 Dec 2;19(12):e0308147. doi: 10.1371/journal.pone.0308147 (PMC11611208; doi:10.1371/journal.pone.0308147)
Supplement: S3 Table — (PDF) [file pone.0308147.s003.pdf]

S3 Table. Relative position between Swedish universities, based on various indicators.

Swedish university	Scholarly Output			Rank Scholarly Output			FWCI			Rank FWCI			Number of researchers			Increase researchers (%)		
	1996 - 2001	2002 - 2011	2012 - 2021	1996 - 2001	2002 - 2011	2012 - 2021	1996 - 2001	2002 - 2011	2012 - 2021	1996 - 2001	2002 - 2011	2012 - 2021	1992 - 2001	2002 - 2011	2012 - 2021	2011 v 2001	2021 v 2011	study period
Karolinska Institutet	19,846	43,059	74,984	*1*	*1*	*1*	1.65	1.83	2.11	*2*	*1*	*1*	16,666	19,423	25,825	*17%*	*33%*	*55%*
Lund University	18,604	42,028	64,353	*2*	*2*	*2*	1.62	1.74	1.89	*3*	*6*	*6*	14,043	16,466	22,030	*17%*	*34%*	*57%*
Uppsala University	15,808	35,595	61,392	*3*	*3*	*3*	1.62	1.77	1.85	*3*	*4*	*7*	12,469	14,057	19,693	*13%*	*40%*	*58%*
University of Gothenburg	9,658	21,629	43,406	*4*	*5*	*6*	1.49	1.60	1.97	*9*	*9*	*3*	9,644	11,253	15,554	*17%*	*38%*	*61%*
KTH Royal Institute of Technology	7,973	25,351	50,001	*6*	*4*	*4*	1.43	1.60	1.76	*12*	*9*	*9*	4,997	8,504	13,978	*70%*	*64%*	*180%*
Stockholm University	6,703	19,853	43,738	*8*	*6*	*5*	1.54	1.76	1.99	*7*	*5*	*2*	5,433	7,732	12,636	*42%*	*63%*	*133%*
Linköping University	6,735	16,877	28,767	*7*	*8*	*8*	1.50	1.57	1.76	*8*	*11*	*9*	5,006	6,848	9,754	*37%*	*42%*	*95%*
Umeå University	6,324	15,501	24,814	*9*	*9*	*9*	1.55	1.69	1.81	*6*	*7*	*8*	4,947	6,426	8,666	*30%*	*35%*	*75%*
Chalmers University of Technology	8,106	17,023	28,817	*5*	*7*	*7*	1.33	1.47	1.57	*14*	*14*	*12*	4,551	5,870	8,633	*29%*	*47%*	*90%*
Swedish University of Agricultural Sciences	5,074	11,389	19,374	*10*	*10*	*10*	1.34	1.50	1.64	*13*	*13*	*11*	3,852	4,821	6,291	*25%*	*30%*	*63%*
Luleå University of Technology	1,512	4,494	10,807	*11*	*11*	*11*	1.31	1.24	1.47	*15*	*18*	*15*	1,044	1,874	3,180	*80%*	*70%*	*205%*
Örebro University	315	4,402	9,837	*16*	*12*	*12*	1.57	1.79	1.97	*5*	*3*	*3*	253	1,603	2,975	*534%*	*86%*	*1076%*
Linnaeus University	368	3,036	6,581	*14*	*13*	*13*	1.24	1.64	1.50	*16*	*8*	*13*	242	1,089	2,048	*350%*	*88%*	*746%*
Malmö University	323	1,665	4,674	*15*	*16*	*14*	1.47	1.30	1.45	*10*	*16*	*17*	267	683	1,618	*156%*	*137%*	*506%*
Mälardalen University	187	1,613	4,525	*18*	*17*	*15*	1.08	1.57	1.48	*18*	*11*	*14*	126	646	1,342	*413%*	*108%*	*965%*
Mid Sweden University	370	1,956	3,877	*13*	*14*	*17*	1.12	1.28	1.47	*17*	*17*	*15*	226	809	1,286	*258%*	*59%*	*469%*
Karlstad University	289	1,881	3,929	*17*	*15*	*16*	1.44	1.35	1.43	*11*	*15*	*18*	233	705	1,252	*203%*	*78%*	*437%*
Stockholm School of Economics	539	1,348	1,765	*12*	*18*	*18*	2.03	1.83	1.94	*1*	*1*	*5*	284	432	576	*52%*	*33%*	*103%*

The indicators are: scholarly output (defined as the count of articles, reviews, books, books chapters and conference papers per year), Field Weighted Citation Impact (FWCI) and count of unique researchers. Data source: SciVal for scholarly outputs and FWCI, Researcher mobility tool for number of researchers.
